# Achieving the earliest possible reperfusion in patients with acute coronary syndrome: a current overview

**DOI:** 10.1186/s40560-018-0285-9

**Published:** 2018-03-15

**Authors:** Takahiro Nakashima, Yoshio Tahara

**Affiliations:** Department of Cardiovascular Medicine, National Cerebral and Cardiovascular Center, 5-7-1 Fujishirodai, Suita, 565-8565 Japan

**Keywords:** ACS, STEMI, NSTEMI, PCI

## Abstract

Acute coronary syndrome (ACS) remains one of the leading causes of mortality worldwide. Appropriate management of ACS will lead to a lower incidence of cardiac arrest. Percutaneous coronary intervention (PCI) is the first-line treatment for patients with ACS. PCI techniques have become established. Thus, the establishment of a system of health care in the prehospital and emergency department settings is needed to reduce mortality in patients with ACS. In this review, evidence on how to achieve earlier diagnosis, therapeutic intervention, and decision to reperfuse with a focus on the prehospital and emergency department settings is systematically summarized.

The purpose of this review is to generate current, evidence-based consensus on scientific and treatment recommendations for health care providers who are the initial points of contact for patients with signs and symptoms suggestive of ACS.

## Background

Acute coronary syndrome (ACS) remains one of the leading causes of mortality worldwide. Appropriate management of this disease will lead to a reduced incidence of cardiac arrest. One major focus of research worldwide is improving outcomes in patients with ACS. In 2015, the Japan Resuscitation Council (JRC) guidelines were updated based on the 2015 International Consensus on Cardiopulmonary Resuscitation and Cardiovascular Care Science with Treatment Recommendations (CoSTR). CoSTR is a systematic and explicit approach to making judgments about the quality of evidence and strength of recommendations. The purpose of this review is to generate current, evidence-based consensus on scientific and treatment recommendations for health care providers who are the initial point of contact for patients with signs and symptoms suggestive of ACS based on the 2015 JRC guidelines.

## Review

### Primary health care algorithm for ACS

**Fig. 1:**
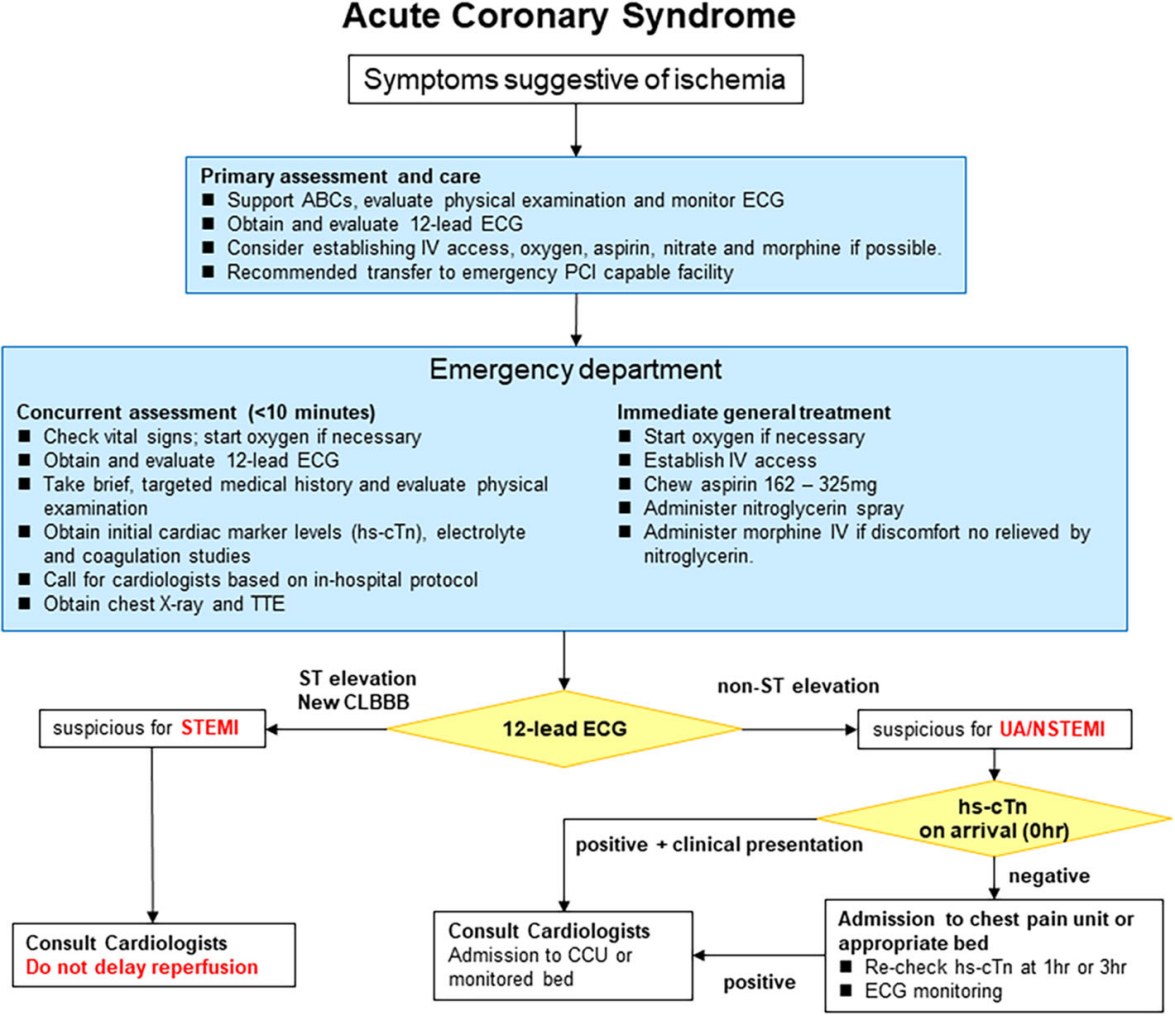
Primary health care algorithm for acute coronary syndrome. *ABC* airway, breathing, and circulation; *CCU* cardiac care unit; *CLBBB* complete left bundle block; *ECG* electrocardiogram; *EMS* emergency medical services; *hs-cTn* high-sensitivity cardiac troponin; *IV* intravenous; *MI* myocardial infarction; *PCI* percutaneous coronary intervention; TTE transthoracic echocardiography; *UA* unstable angina

Figure 1 shows the primary algorithm for ACS. In patients presenting to the emergency department (ED) with chest pain of suspected cardiac etiology, prompt diagnosis and treatment of ACS are the key concepts. The urgency and severity of ACS are evaluated using the history and physical examination in the ED. Twelve-lead electrocardiogram (ECG) plays a central role in the triage process. For patients with ST-elevation myocardial infarction (STEMI), the physician cooperates with the cardiologist to prioritize revascularization. On the other hand, for patients with no ST elevation but non-STEMI (NSTEMI) or high-risk unstable angina is suspected, the emergency physician and cardiologist should work together on cardiac care unit admission. These patients have a high rate of adverse cardiac events (death, nonfatal myocardial infarction, and urgent revascularization). Thus, an invasive strategy such as percutaneous coronary intervention (PCI) is often selected in addition to medical therapy. In patients with suspected ACS, normal initial biomarkers and nonischemic ECG, 0 h/1 h or 0 h/3 h rule-out algorithm of NSTEMI using high-sensitivity cardiac troponin (hs-cTn) may be recommended as a safe and effective strategy in the ED (see the “Biomarkers in ACS” section). Transthoracic echocardiography is helpful not only in the evaluation of wall motion abnormality, left ventricular function, and mechanical complications such as ventricular free wall rupture, ventricular septal perforation, and papillary muscle rupture, but also in the diagnosis of conditions such as acute aortic dissection, acute pulmonary embolism, and acute pericarditis. Chest X-ray is helpful in diagnosing and assessing the severity of ACS, but is not always necessary if ACS is strongly suspected and obtaining a chest X-ray will delay revascularization. Furthermore, waiting for the results of laboratory data to diagnose ACS should not cause delay in revascularization. Time from hospital arrival to transport to facilities capable of performing emergency PCI capable should be within 30 min.

### Diagnostic interventions in ACS

#### Risk stratification in ACS

Various patient demographic factors might impede seeking medical help rapidly and add to further in-hospital treatment delay. Many reports have suggested that older age, female gender, racial or ethnic minority status, low socioeconomic status, and residing alone are independent factors associated with in-hospital treatment delay [1, 2]. Providers should be trained to expeditiously identify patients with ACS irrespective of age, gender, socioeconomic status, or living arrangement. On the other hand, signs and symptoms may be useful in combination with other important information such as biomarkers, risk factors, ECG, and other diagnostic test results, in triaging and making some treatment and investigational decisions for ACS in the out-of-hospital and ED settings. The Global Registry of Acute Coronary Events (GRACE) score provides accurate stratification of risk on admission and discharge (Table 1) [3, 4].

**Table 1:** The Global Registry of Acute Coronary Events (GRACE) score

	Score		Score		Score		Score		Score		Score
Age (year)		Heart rate (bpm)		Systolic BP (mmHg)		Killip class		Creatinine (mg/dL)			
< 40	0	< 70	0	< 80	63	Class I	0	0.0–0.39	2	Cardiac arrest at admission	43
40–49	18	70–89	7	80–99	58	Class II	21	0.4–0.79	5		
50–59	36	90–109	13	100–119	47	Class III	43	0.8–1.19	8	Elevated cardiac markers	15
60–69	55	110–149	23	120–139	37	Class IV	64	1.2–1.59	11		
70–79	73	150–199	36	140–159	26			1.6–1.99	14	ST-segment deviation	30
80 <	91	200 <	46	160–199	11			2.0–3.99	23		
				200 <	0			4.0 <	31		

In-hospital mortality: low risk (≤ 10), intermediate risk (109–140), and high risk (> 140) are < 1%, 1–3%, and > 3%, respectively. Post-discharge to 6 months death: low risk (≤ 88), intermediate risk (89–118), and high risk (> 8) are < 3%, 3–8%, and > 8%, respectively

*BP* blood pressure

#### ECG

The ECG is essential for the initial triage and initiation of management in patients with possible ACS, especially in the ED and out-of-hospital settings. Many observational studies have shown the benefit of prehospital 12-lead ECG in reducing 30-day mortality, first-medical contact-to-reperfusion time, door-to-balloon time, and door-to-needle time compared with no ECG in patients with STEMI [5–13]. The 2015 JRC guidelines recommend prehospital 12-lead ECG acquisition with hospital notification for adult patients with suspected STEMI (strong recommendation, low-quality evidence). However, prehospital 12-lead ECG is not currently widespread in Japan. Thus, we should consider the use of prehospital 12-lead ECG in order to start specific therapy for STEMI more quickly. At the same time, we need to develop a computer-assisted ECG interpretation system for STEMI and an educational program for nurses and paramedics in ECG interpretation for STEMI [14–18].

#### Biomarkers in ACS

Some observational studies have shown that hs-cTn is helpful for excluding the diagnosis of ACS [19–26]. The 2015 JRC guidelines recommend against using only hs-cTnT and hs-cTnI measured at 0 and 2 h to rule out ACS (strong recommendation, very low-quality evidence). However, in low-risk patients (as defined by the Vancouver rule or a Thrombolysis in Myocardial Infarction Trial [TIMI] score of 0 or 1), the guidelines suggest that negative hs-cTnI at 0 and 2 h and negative hs-cTnI or hs-cTnT at 0 and 3–6 h may be used to rule out ACS (weak recommendation, low-quality evidence). Further studies are needed to evaluate the combination of troponins and clinical risk scores to determine which patients with chest pain may be safely discharged from the ED.

#### Imaging techniques

Noninvasive tests such as cardiac computed tomography (CT), cardiac magnetic resonance (MR), myocardial perfusion imaging, and echocardiography may be considered in selected patients who present to the ED with chest pain and an initial nondiagnostic conventional work-up that included 12-lead ECG or cardiac biomarkers. It is reasonable to consider both radiation and iodinated contrast exposure when using cardiac CT and myocardial perfusion imaging. Moreover, in some low-risk patients, these noninvasive tests may decrease cost, length of stay, and time to diagnosis [27–29]. They might provide valuable short-term and long-term prognostic information on future major cardiac events. However, there are insufficient data to assess the impact of imaging techniques on mortality. A combination of these techniques and chest pain observation units may be useful, and the spread of chest pain observation units is expected in Japan.

### Therapeutic interventions for ACS

#### Oxygen therapy

Some randomized controlled trials (RCTs) have shown no difference between no oxygen and supplementary oxygen administration with regards to mortality (odds ratio [OR], 0.91; 95% confidence interval [CI], 0.25–3.34) [30–34]. The 2015 JRC guidelines suggest withholding routine high-concentration oxygen supplementation (8 L/min) in normoxic (SpO_2_ > 93%) patients with ACS (weak recommendation, very low-quality evidence), except for patients with previous myocardial infarction, severe chronic obstructive pulmonary disease, respiratory failure, cardiogenic shock, central cyanosis, SpO_2_ < 85%, or dyspnea from any other cause. Moreover, two recent RCTs show that routine supplementary oxygen administration is not beneficial [33, 35]. However, there is lack of evidence regarding low-concentration oxygen supplementation.

#### Nitroglycerin

Although it is reasonable to consider early administration of nitroglycerin in selected patients without contraindications, insufficient evidence exists to support or refute the routine administration of nitroglycerin in the ED or prehospital setting in patients with suspected ACS. There may be some benefit if nitroglycerin results in pain relief. When non-cardiologist physicians administer nitroglycerin, they give one sublingual nitroglycerin tablet or spray every 3 to 5 min, which may be repeated a total of 3 times if the patient remains hemodynamically stable. If right ventricular (RV) infarction is suspected, vasodilators, including nitroglycerin, are contraindicated because hemodynamic status with RV infarction depends on RV filling pressure. Relief of chest discomfort with nitroglycerin is neither sensitive nor specific for ACS; gastrointestinal etiologies as well as other causes of chest discomfort can respond to nitroglycerin administration.

#### Analgesics and sedation

Morphine can relieve chest pain, alleviate the work of breathing, reduce anxiety, and favorably affect ventricular loading conditions [36]. Despite limited direct evidence to support or refute the practice, morphine should be administered intravenously and titrated to pain relief in patients with STEMI. Morphine may be considered for pain relief in patients with suspected NSTEMI. Physicians give patients morphine 2 to 4 mg via intravenous injection, which may be increased to 8 mg every 5 to 15 min if it is not effective [37]. Other forms of analgesia (e.g., buprenorphine 0.1 to 0.2 mg) should be considered for patients with active chest discomfort. While anxiolytics may be administered to patients with ACS to alleviate anxiety, there is no evidence that anxiolytics facilitate ECG resolution, reduce infarct size, or decrease mortality in patients with suspected ACS. Non-steroidal anti-inflammatory drugs (NSAIDs) should not be administered because they may be harmful in patients with suspected ACS. Some studies have shown that NSAIDs are associated with an increased risk of mortality, reinfarction, hypertension, heart failure, and myocardial rupture in patients with STEMI [38, 39]. Patients with suspected ACS who are taking NSAIDs should have them discontinued when feasible.

#### Aspirin (acetylsalicylic acid) and adenine diphosphate (ADP) receptor antagonists

Despite limited direct evidence to support or refute the practice [40], the 2015 CoSTR guidelines mentioned that it may be reasonable to consider aspirin as soon as possible, without a history to exclude a true allergy or bleeding disorder. Moreover, some RCTs have shown that compared with in-hospital administration, there is no additional benefit with prehospital administration of an ADP receptor antagonist in terms of 30-day mortality (OR, 1.58; 95% CI, 0.90–2.78) and major bleeding (OR, 1.12; 95% CI, 0.72–1.74) [41–43]. These studies suggest that ADP receptor antagonists can be given to patients with suspected STEMI and planned primary PCI in either the prehospital or the in-hospital setting (very low-quality evidence, weak recommendation). However, in Japan, administration of aspirin for suspected STEMI outside of the hospital by emergency medical service (EMS) personnel is prohibited by law. When a primary PCI approach is being planned, physicians can give patients aspirin (162 to 325 mg) and ADP receptor antagonists (clopidogrel 300 mg or prasugrel 20 mg). Further investigation is needed to confirm the benefit of prehospital aspirin and ADP receptor antagonist administration in the doctor car or medical helicopter.

#### Anticoagulants

In patients with suspected out-of-hospital STEMI, a non-RCT showed no benefit of prehospital unfractionated heparin (UFH) on 30-day mortality compared with in-hospital UFH (OR, 1.07; 95% CI, 0.595–1.924) [44]. The 2015 CoSTR guidelines suggest that UFH administration can occur in either the prehospital or in-hospital setting in patients with suspected STEMI and a planned primary PCI approach. There is insufficient evidence to change existing practice (weak recommendation, very low-quality evidence). However, in Japan, EMS personnel cannot administer anticoagulants in the prehospital setting. Further investigation is needed to confirm the benefit of prehospital fibrinolysis in the doctor car or medical helicopter. Physicians administer UFH as a single intravenous injection with a target activated clotting time (ACT) of > 250 s. We note that most evidence about UFH in patients ACS were from the pre-primary PCI era. Further investigation is needed to approve prehospital anticoagulant administration by EMS personnel and the use of enoxaparin for STEMI in Japan.

### Reperfusion decisions in patients with STEMI

The 2015 JRC guidelines address the question of which reperfusion strategy is best under specific circumstances. The options available for reperfusion will depend on the local prehospital system and availability of PCI centers. They consider reperfusion decisions in relation to regional availability (e.g., prehospital fibrinolysis versus ED or prehospital fibrinolysis versus direct transport to PCI). Table 2 shows the most appropriate reperfusion strategy by time from symptom onset and anticipated treatment delay.

**Table 2:** Most appropriate reperfusion strategy by time from symptom onset and anticipated treatment delays

Treatment delay	Time from symptom onset		
	< 2 h	2–3 h	3–6 h*
< 60 min	Primary PCI	Primary PCI or fibrinolysis^†^	Primary PCI
60–120 min	Fibrinolysis^†^	Primary PCI or fibrinolysis^†^	Primary PCI
> 120 min	Fibrinolysis^†^	Fibrinolysis^†^	Fibrinolysis^†^

Patients with higher risk, including those with Killip class > 1, may benefit from primary PCI even when there are treatment delays up to 120 min

*PCI* percutaneous coronary intervention

*If time from symptom onset is greater than 6 h, primary PCI is appropriate regardless of treatment delay

^†^In case of fibrinolytic therapy, immediate transfer to a PCI center after fibrinolysis should be considered for cardiac angiography within 3 to 24 h

#### Prehospital fibrinolysis versus ED fibrinolysis

Some RCTs have shown that prehospital fibrinolysis reduced in-hospital mortality without increasing intracranial hemorrhage and bleeding compared with in-hospital fibrinolysis (OR, 0.46; 95% CI, 0.23–0.93) [44–47]. When fibrinolysis is the planned treatment strategy, the 2015 JRC guidelines recommend prehospital fibrinolysis over in-hospital fibrinolysis for STEMI in health systems where typical transport time is greater than 30 min and prehospital fibrinolysis can be accomplished by a physician in the ambulance or medical helicopter with well-established protocols, comprehensive training programs, and quality assurance programs in place (strong recommendation, moderate-quality evidence).

#### Prehospital triage to a PCI center versus prehospital fibrinolysis

There is moderate-quality evidence that mortality is not reduced and low-quality evidence of harm from fibrinolysis [48, 49]. The 2015 JRC guidelines suggest that direct triage and transport for PCI is preferred in geographic regions where PCI facilities are not available (weak recommendation, low-quality evidence). On the other hand, the 2015 CoSTR suggest that prehospital fibrinolysis is a reasonable alternative to triage and direct transport to a PCI center in geographic regions where PCI facilities are not available. In Japan, prehospital fibrinolysis is preferred but a physician must be present because only physicians can perform fibrinolysis. Further investigation is needed to confirm the benefit of prehospital fibrinolysis in the doctor car or medical helicopter.

#### Delayed PCI versus fibrinolysis stratified by time since symptom onset

Some RCTs have shown that compared with fibrinolysis, delayed PCI is associated with higher 30-day mortality (OR, 2.6; 95% CI, 1.2–5.64) and 5-year mortality (OR, 2.03; 95% CI, 1.1–5.64) [50, 51]. In patients with STEMI presenting less than 2 h after symptom onset for whom primary PCI will result in a delay of greater than 60 min, the 2015 JRC guidelines suggest fibrinolysis over primary PCI (weak recommendation, low-quality evidence) [49, 52, 53]. Further investigation on delayed PCI versus fibrinolysis is needed.

#### ED fibrinolysis, transport only for rescue PCI, routine early angiography, transport for PCI or only rescue PCI

In adult patients with STEMI in the ED of a hospital without PCI capabilities, some RCTs have shown that transfer without fibrinolysis to a PCI center for angiography is associated with lower 30-day mortality compared with immediate in-hospital fibrinolysis and only transfer for ischemia-driven PCI in the first 24 h (OR, 0.66; 95% CI, 0.50–0.86) [54, 55]. For adult patients presenting with STEMI in the ED of a hospital not capable of performing PCI, the 2015 JRC guidelines recommend emergency transfer without fibrinolysis to a PCI center as opposed to immediate in-hospital fibrinolysis and transfer only for rescue PCI (strong recommendation, moderate-quality evidence). On the other hand, some RCTs have shown no difference in 30-day mortality between immediate in-hospital fibrinolysis and routine transfer for angiography compared with transfer to a PCI center (OR, 0.84; 95% CI, 0.24–2.98) [49, 56]. They suggest fibrinolytic therapy with routine transfer for angiography as an alternative to immediate transfer to PCI (weak recommendation, very low-quality evidence). Some RCTs have shown no difference in 30-day and 1-year mortality between either immediate in-hospital fibrinolysis and routine transfer for angiography at 3 to 6 h (or up to 24 h) and immediate in-hospital fibrinolysis and only transfer for ischemia-driven PCI (rescue PCI) (OR, 0.96; 95% CI, 0.64–1.44, OR 0.54; 95% CI, 0.16–1.89, respectively) [49, 57, 58]. Thus, for patients with STEMI who underwent ED fibrinolysis when primary PCI was not available on-site, the 2015 JRC guidelines suggest transport for early routine angiography in the first 3 to 6 h (or up to 24 h) rather than only transport for ischemia-guided angiography (weak recommendation, moderate-quality evidence).

The current evidence indicates that PCI from 3 to 24 h after fibrinolysis reduces reinfarction. The optimal timing within this time window has not been established. Similarly, the optimal management is unclear for patients after fibrinolysis in remote areas where transport to PCI is difficult or prolonged [54, 58–64].

### Medications for ACS

To reduce the incidence of major adverse cardiac event and improve long-term survival, some additional medical therapies have been proposed. However, most of the data supporting the use of these therapies were gathered from patients after admission. To date, there is no evidence on which additional medical therapies in the prehospital or ED setting are important for patients with ACS.

#### Antiarrhythmics

Avoiding preventive administration of antiarrhythmics is reasonable in patients with ACS.

#### β-blockers

Avoiding routine intravenous administration of β-blockers during the initial prehospital or ED evaluation is reasonable for patients with ACS. For patients with ACS, there is no evidence to support routine intravenous administration of β-blockers during the initial prehospital or ED evaluation. Intravenous administration of β-blockers may be reasonable for selected patients with severe hypertension and tachycardia [65, 66]. On the other hand, contraindications to β-blockers include moderate to severe left ventricular failure, pulmonary edema, bradycardia, and hypotension. The effect of early β-blocker administration has not been fully studied in the primary PCI era.

After the patient is stabilized, starting an oral agent of β-blocker at a low dose before discharge is reasonable [67]. A recent multicenter registry of AMI in the PCI era has shown that β-blockers are associated with reduced mortality during long-term follow-up [68].

#### Angiotensin-converting enzyme inhibitors (ACE-Is) and angiotensin II receptor blockers (ARBs)

ACE-I and ARB administration after admission is known to reduce mortality in patients with acute myocardial infarction [69, 70]. However, there is insufficient evidence to support the routine administration of ACE-Is and ARBs in the prehospital and ED settings.

#### HMG-CoA reductase inhibitors (statins)

Statin therapy for patients with ACS soon after admission is reasonable in patients without contraindications [71]. Statins should be continued for patients with ACS who are already being treated with statins [72].

### Hospital reperfusion decisions after return of spontaneous circulation (ROSC)

#### PCI after ROSC with or without ST elevation

After ROSC, some observational studies have shown that emergency cardiac catheterization in patients with ST elevation is associated with increased in-hospital survival (OR, 0.35; 95% CI, 0.31–0.41) and favorable neurological survival (OR 2.54; 95% CI, 2.17–2.99) compared with catheterization laboratory evaluation later in the hospital stay or no catheterization [73–76]. On the other hand, after ROSC in patients without ST elevation, two observational studies have shown the benefit of emergency cardiac catheterization on in-hospital mortality (OR, 0.51; 95% CI, 0.35–0.73) and favorable neurological survival (OR 1.96; 95% CI, 1.35–2.85) compared with catheterization laboratory evaluation later in the hospital stay or no catheterization [73, 76]. Thus, the 2015 JRC guidelines recommend emergency cardiac catheterization laboratory evaluation rather than cardiac catheterization later in the hospital stay or no catheterization in selected adult patients with ROSC after out-of-hospital cardiac arrest of suspected cardiac origin with ST elevation (strong recommendation, low-quality evidence) or without ST elevation on ECG (weak recommendation, very low-quality evidence). In patients with ST elevation, a variety of factors were more likely to be associated with cardiac catheterization: male gender, younger age, ventricular fibrillation as the presenting cardiac arrest rhythm, witnessed arrest, bystander cardiopulmonary resuscitation (CPR), and being supported with vasopressors or left ventricular assist devices. Patient characteristics that were less likely to be associated with angiography were diabetes mellitus, renal failure, and heart failure. On the other hand, in patients without ST elevation, a variety of factors such as patient age, CPR duration, hemodynamic instability, presenting cardiac rhythm, neurologic status upon hospital arrival, and perceived likelihood of cardiac etiology influenced the decision for intervention. Further investigation is needed to confirm the benefit seen in the first two observational studies. Ideally, randomized studies would help identify if there are certain subgroups of patients that would benefit more from angiography after ROSC.

#### Mechanical support for ACS with cardiogenic shock or cardiac arrest

ACS patients are often hemodynamically unstable. Management of these patients can be challenging. The use of mechanical support is taken into consideration for ACS patients with cardiogenic shock, defined as systolic blood pressure of less than 90 mmHg, use of catecholamines to maintain a systolic pressure of at least 90 mmHg, clinical signs of pulmonary congestion, or signs of impaired organ perfusion. In ACS patients with shock, use of an intra-aortic balloon pump (IABP), percutaneous left ventricular support device (Impella®, Abiomed, Danvers, Massachusetts), or veno-arterial extracorporeal membrane oxygenation (VA-ECMO) can be considered. Although the American Heart Association and European Society of Cardiology guidelines have downgraded the use of IABP [36, 77], the Japanese Cardiology Society guidelines give the use of IABP for cardiogenic shock a class I recommendation because percutaneous left ventricular support device (Impella®) was not yet approved in Japan at the time. Percutaneous left ventricular support device (Impella®) has been approved in Japan since 2017. Further accumulation of clinical data in Japan is needed. On the other hand, the 2015 JRC guidelines suggest that VA-ECMO is a reasonable rescue therapy for selected patients with cardiac arrest refractory to conventional CPR (weak recommendation, very low-quality evidence) [78, 79]. In patients with cardiac arrest due to ACS, VA-ECMO may allow providers additional time to treat acute coronary artery occlusion [80]. However, these techniques require adequate vascular access and specialized equipment.

### Health care system interventions for ACS

#### Prehospital notification for activation of the cardiac catheterization laboratory and calling for the catheterization team

To prepare for primary PCI, the 2015 JRC guidelines recommend prehospital notification to activate the cardiac catheterization laboratory and calling for the catheterization team (strong recommendation, very low-quality evidence). Some observational studies have shown that prehospital activation of the catheterization team reduces 30-day mortality (OR, 0.41; 95% CI, 0.30–0.56) [7, 10, 81]. Establishment of a health care system in the prehospital and ED settings is needed (Table 3).

**Table 3:** Ways to improve systems of care for acute coronary syndrome

■ Emergency physician calls for the catheterization team ■ Single call to a central operator ■ Real-time data feedback ■ Institutional commitment ■ Team-based approach ■ Catheterization team arrival within 20 min of being called ■ Having an interventional cardiologist immediately available at the hospital	

It is reasonable for hospitals to consider these measures to improve systems of care for acute coronary syndrome

## Conclusion

**Fig. 2:**
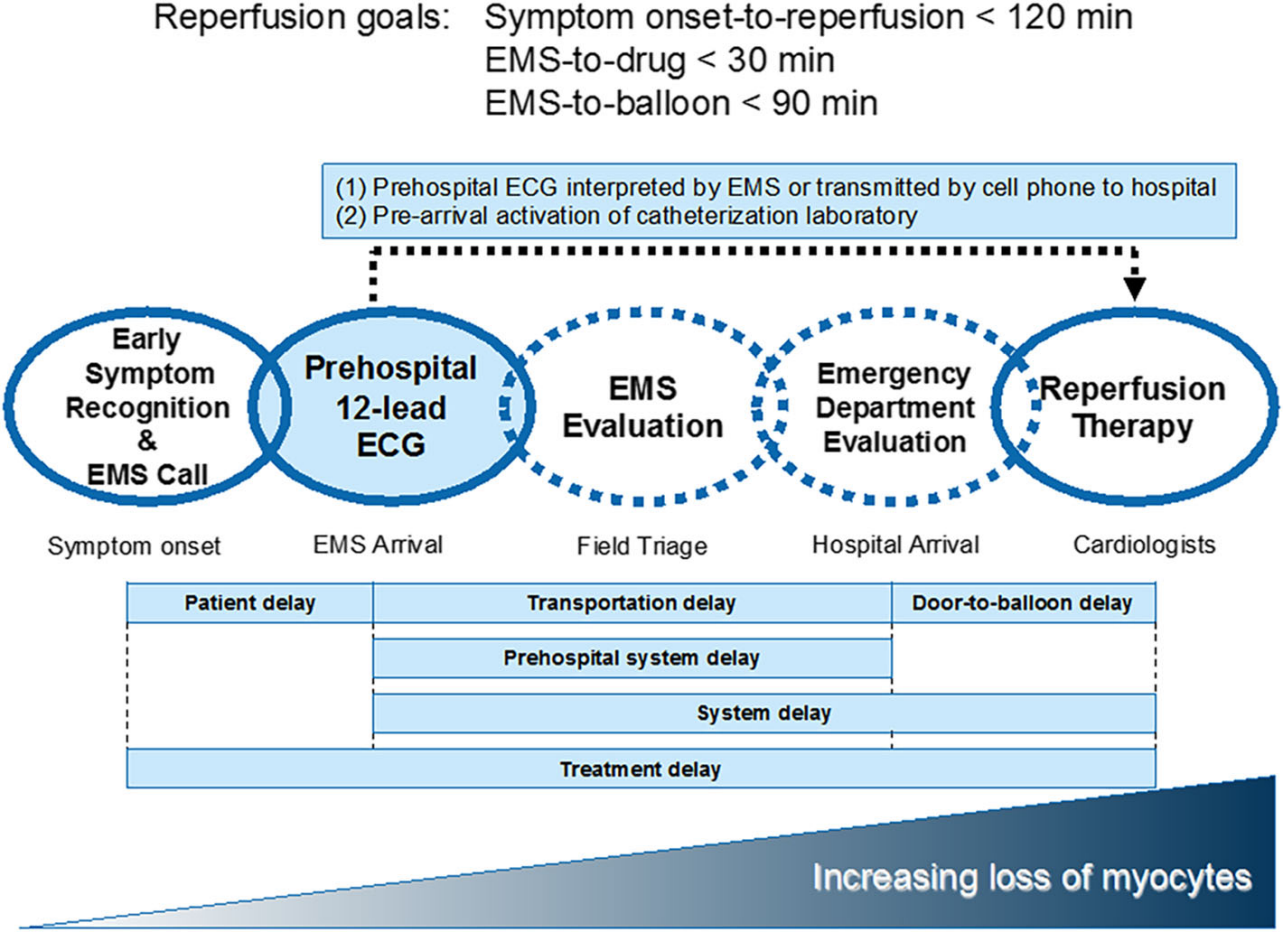
Time-course goals for reperfusion in acute coronary syndrome. The target time from symptom onset to reperfusion is ≤ 120 min. The target time from first medical contact to fibrinolysis is ≤ 30 min. The target time from first medical contact to percutaneous coronary intervention is ≤ 90 min. However, there are many factors that can delay reperfusion. To prevent delay, we must educate citizens to call EMS as soon as symptoms occur. To prevent transportation, prehospital system, and door-to-balloon delays, prehospital 12-lead ECG is recommended. Prehospital ECG can shorten the duration of EMS evaluation (hospital selection) and emergency department evaluation (decision to reperfuse). *ECG* electrocardiogram, *EMS* emergency medical services

Several systems-related strategies have been developed to improve quality of care for patients with ACS and reduce reperfusion delays for patients with STEMI. Some strategies that focus on patients identified as having ACS in the prehospital and ED settings (Fig. 2) include the use of prehospital 12-lead ECG and time-saving strategies to facilitate early diagnosis and rapid treatment for patients with STEMI. Recently, PCI technique has become established. Thus, we must construct a system of health care to achieve early reperfusion in the prehospital and ED settings to reduce mortality in patients with ACS.

## Abbreviations

ACE-Is: Angiotensin-converting enzyme inhibitors
ACS: Acute coronary syndrome
ACT: Activated clotting time
ARBs: Angiotensin II receptor blockers
CoSTR: Consensus on Cardiopulmonary Resuscitation and Cardiovascular Care Science with Treatment Recommendations
CPR: Cardiopulmonary resuscitation
CT: Computed tomography
ECG: Electrocardiogram
ED: Emergency department
EMS: Emergency medical service
GRACE: Global Registry of Acute Coronary Events
hs-cTn: High-sensitivity cardiac troponin
IABP: Intra-aortic balloon pump
JRC: Japan Resuscitation Council
MR: Magnetic resonance
NSAID: Non-steroidal anti-inflammatory drug
NSTEMI: Non-ST-elevation myocardial infarction
PCI: Percutaneous coronary intervention
ROSC: Return of spontaneous circulation
RV: Right ventricular
STEMI: ST-elevation myocardial infarction
TIMI: Thrombolysis in Myocardial Infarction Trial
UFH: Unfractionated heparin
VA-ECMO: Veno-arterial extracorporeal membrane oxygenation
